# A Novel Method for Detecting Ferromagnetic Wear Debris with High Flow Velocity

**DOI:** 10.3390/s22134912

**Published:** 2022-06-29

**Authors:** Feng Wang, Zhijian Liu, Xiaojing Ren, Sen Wu, Meilin Meng, Yulin Wang, Xinxiang Pan

**Affiliations:** 1 College of Marine Engineering, Dalian Maritime University, Dalian 116026, China; wangfeng6633@163.com (F.W.); dlmuwusen@163.com (S.W.); mml2220200039@dlmu.edu.cn (M.M.); wyl001218@163.com (Y.W.); dmupanxx@gmail.com (X.P.); 2 College of Foreign Languages, Dalian Maritime University, Dalian 116026, China; gracerxj@dlmu.edu.cn; 3 College of Navigation, Guangdong Ocean University, Zhanjiang 524088, China

**Keywords:** inductance detection, wear debris, particles velocity, high-frequency voltage acquisition (HFVA)

## Abstract

Inductance detection is an important method for detecting wear debris in ship lubricating oil. Presently, an LCR (inductance, resistance, capacitance) meter is generally used to detect wear debris by measuring the inductance change of the sensing coil. When ferromagnetic debris passes through the sensing coil, a pulse will appear in the inductance signal. Previous studies have shown that the amplitude of the inductance pulse decreases significantly with the increase in the particles’ velocity. Therefore, it is difficult to detect ferromagnetic debris with a high flow velocity using an LCR meter. In this paper, a novel method, high-frequency voltage acquisition (HFVA), is proposed to detect ferromagnetic debris. Different from previous methods, the wear debris was detected directly by measuring the voltage change of the sensing coil, while the synchronized sampling method was utilized to ensure the higher-frequency acquisition of the sensor output signal. The experimental results show that when the velocity of particles increased from 6 mm/s to 62 mm/s, the amplitude of the signal pulse obtained by HFVA decreased by only 13%, which was much lower than the 85% obtained by utilizing the LCR method.

## 1. Introduction

At present, the total number of ships around the world continues to increase along with global trade development, which generates higher demand for condition monitoring of ship equipment [1,2]. Usually, the monitoring method of mechanical equipment is mainly based on the monitoring of a series of working parameters, such as vibration [3,4,5], sound [6], temperature [7], pressure [8], and flow rate [9]. However, in addition to the above parameters, wear debris in lubricating oil also contains a lot of information about equipment operations, which is of great importance to the condition monitoring and fault diagnosis of mechanical equipment. Research shows that more than 75% of hydraulic machine failures, about 40% of rolling bearing failures, 38.5% of gear failures, and 35% of diesel engine failures are caused by wear debris [10]. During the normal running life of a machine, the concentration of wear debris that has fallen off due to the friction pair remains at a low level, and its size stays below 20 μm. However, when abnormal wear begins, the concentration of wear debris increases quickly and its size grows up to 50–100 μm [11,12,13]. Therefore, the concentration and size of wear debris could indicate the degree and process of wear [14]. In addition, non-ferrous metal materials are usually used as a coating on the friction pair for reducing the contact friction, which enables the wear position and the failure of mechanical components to be determined according to the nature of wear debris in the lubricating oil [10]. Therefore, we can obtain the running information of ship machinery by detecting the wear debris in ship lubricating oil [15]. Thus, the detection of wear debris is meaningful for marine mechanical failure prediction and diagnosis [16,17,18]. A series of methods based on optics [19,20,21,22], acoustics [23,24], capacitance [25,26,27], and inductance [10,28,29,30] have been proposed for wear debris detection. The optical method has high detection accuracy. However, it is easily affected by oil transparency and air bubbles [31]. The acoustic method can distinguish air bubbles from solid wear debris. Nevertheless, it can be affected by oil viscosity, flow velocity, and mechanical vibration, which can make it difficult to be put into application [32]. The capacitance method can distinguish metal particles from bubbles according to the difference in the dielectric constant, but it cannot differentiate ferrous and non-ferrous debris in oil [10]. The inductance detection method is a non-destructive method based on the principle of electromagnetic induction. This method can not only distinguish the properties of wear debris but also has a strong adaptability to oil quality and the environment, making it widely used in wear debris detection [33]. There are many different methods for inductance detection of ferromagnetic particles, which can additionally be divided into voltage output and inductance output. Voltage-type output sensors generally consist of multiple coils including excitation coils and induction coils. When ferromagnetic particles pass through, the induced electromotive force of the induction coil will change, and the sensor will output a voltage pulse [34]. This type of sensor often has high sensitivity and throughput [35]. However, this method is also easily affected by the flow velocity of ferromagnetic particles. Further, an inductive output sensor can work with only one coil. It is mainly based on the inductance change caused by the ferromagnetic particles of the output. This type of sensor usually has a simpler circuit structure and a simpler manufacturing process, but its detection throughput is usually low [36,37]. For the inductance detection method, many researchers have made a great effort to improve the throughput. Du et al. [38] proposed a multiplexed, multichannel, inductive pulse sensor based on resonant frequency division multiplexing to raise the throughput. Wu et al. [39] presented a novel multichannel wear debris sensor based on phase division multiplexing, which could simultaneously detect wear debris in four channels without increasing the number of excitation sources and data acquisition instruments. Du et al. [13] presented a high-throughput inductive pulse sensor based on the inductive Coulter counting principle. In this instance, a high throughput was achieved by using a two-layer planar coil with a meso-scale fluidic pipe crossing the coil’s center. Shi et al. [40] proposed an impedance debris sensor based on a high-gradient magnetic field for high throughput. The detection throughput was increased, through which the square channel could make full use of the sensitive detection region. Briefly, the methods mentioned above employ a multichannel or a large cross-sectional area pipe to increase the throughput, although this makes the detection system more complex.

However, there is another way to improve the throughput, that is, increasing the flow velocity of the wear debris. Unfortunately, the increased velocity of the wear debris would make it difficult for it to be detected with traditional methods. Wang et al. [36] proposed that the amplitude of the induction pulse is negatively proportional to the particles’ velocity under the LCR meter detection. Their experimental results showed that when the velocity of the particles was increased from 1.54 mm/s to 5.95 mm/s, the inductance variation of the coil decreased by up to 36.5%, from 91.4 nH to 58.0 nH. In order to detect high-velocity particles without decreasing the signal amplitude, a novel detection method, high-frequency voltage acquisition (HFVA), is presented in this paper. This method is mostly based on a high-frequency synchronized sampling of the coil voltage, has a higher sampling frequency and a faster sampling speed, and can collect more samples at the same time. The negative effect of the particle velocity on the pulse signal can be significantly weakened. We experimentally studied the variation in the induction pulse amplitude at different velocities using 81 μm ferromagnetic particles.

## 2. The Detection Principle

### 2.1. Debris Sensing Mechanism

When a high-frequency alternating voltage is applied to the sensing coil, an alternating magnetic field is generated around the coil [1,41]. The ferromagnetic particles that pass through the sensing coil would be magnetized by the magnetic field. The magnetized debris produces an additional magnetic field in the same direction as the original magnetic field, which greatly increases the strength of the magnetic field [42]. At the same time, an eddy current also generates a magnetic field inside the debris. This generates a magnetic field opposite to the original magnetic field, which weakens the strength of the original magnetic field. However, due to the high permeability of the ferromagnetic debris, the enhancement of the magnetic field strength by the magnetization effect is much greater than the decrease in strength caused by the eddy current effect [43]. Thus, the influence of the eddy current effect on the original magnetic field can be ignored. As a result, the total magnetic field strength increases, along with the magnetic flux that passes through the sensing coil, which in turn makes the inductance of the coil increase [44,45]. Conversely, when non-ferromagnetic debris passes through the sensing coil, the eddy current effect is dominant over the magnetization effect, which reduces the inductance of the sensing coil [46]. When the inductance value changes, the output voltage of the sensing coil changes accordingly. Therefore, wear debris can be detected and differentiated with the inductance or voltage change measurement.

### 2.2. Signal Detection Method

In our work, to prove the effectiveness of the HFVA method, the detection signals were measured by two methods at the same time. One signal was measured by the HFVA method, and another was measured by an LCR meter (under the same conditions). When using an LCR meter for the detection, the LCR meter usually directly obtains the current voltage value and phase angle change of the sensing coil and then converts them into inductance values and outputs [47,48]. This conversion process requires a certain response delay time. According to the literature (http://literature.cdn.keysight.com/litweb/pdf/5989-4435EN.pdf, accessed on 10 June 2022), the measurement time of the LCR meter—under the three measurement modes of “SHORT”, “MED”, and “LONG”—gradually increases with the decrease in frequency. At the highest frequency (2 MHz), the measurement times of the above three measurement modes are 5.6 ms, 88 ms, and 220 ms, respectively. For particles with a high-velocity flow, the time of passing through the detection coil is far less than the above measurement time. Therefore, the LCR meter is unable to accurately trace the inductance change of the sensing coil when the debris moves at a high velocity.

**Figure 1:**
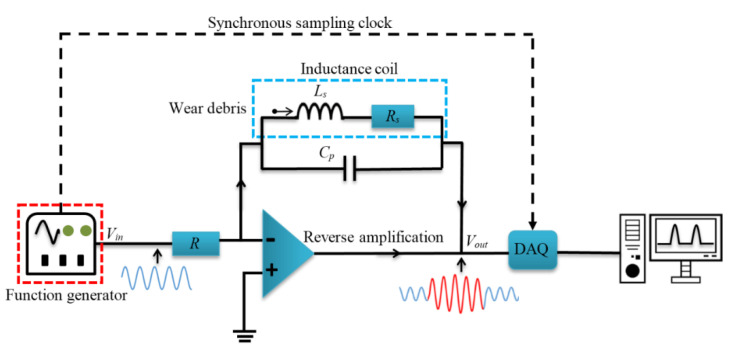
Schematic of signal detection system. Firstly, the excitation signal is sent out by the function generator. After passing through the voltage follower, the signal is applied to the reverse amplifier composed of the inductance coil, operational amplifier, and capacitor, amplified, and output. Finally, the signal is collected by the signal acquisition card and sent to the computer for processing and output.

Contrastingly, in the HFVA method, the debris is detected by measuring the output voltage directly. Without the need for data conversion, the response time of the detection equipment is greatly shortened, which makes the system more sensitive. Figure 1 shows the signal detection system of HFVA. The sensing circuit is composed of a voltage follower, a gain inverting amplifier, and a sensing coil. The sensing coil is connected with capacitance in parallel to form a parallel resonance circuit. Except for the sensing circuit, the whole detection system also contains a function generator, signal acquisition equipment (DAQ), and a computer. The function generator produces a sine wave excitation signal, which is applied to the sensing coil after passing through the voltage follower. When a particle passes through, the output voltage amplitude will increase. Finally, the peaks of the output signal are recorded in real time with the high-frequency synchronized sampling method [49,50,51,52]. The recorded data are processed with the digital low-pass filter in LabVIEW and saved to the computer. The relationship between the output voltage, *V_out_*, and input excitation signal, *V_in_*, is shown in Equation (1):

(1) $$V_{\mathrm{out}}{=-V}_{\mathrm{in}}\times \frac{\frac{\frac{L_{s}}{C_{p}}}{R_{s}+j\left({\mathrm{wL}}_{s}-\frac{1}{{\mathrm{wC}}_{p}}\right)}}{R}{=-V}_{\mathrm{in}}\times \frac{Z}{R}$$

in which *V_in_* is the input excitation voltage, *V_out_* is the output voltage, *C_p_* is the parallel capacitance, *R_s_* is the sensor internal resistance, *L_s_* is the inductance, and *Z* is the impedance of the parallel resonance circuit. The sensor output signal, *V_out_*, is proportional to the impedance, *Z*. Due to the use of a gain inverting amplifier, the polarities of the input and output signals are opposite.

## 3. Experiment Setup

### 3.1. Sample Preparations

**Figure 2:**
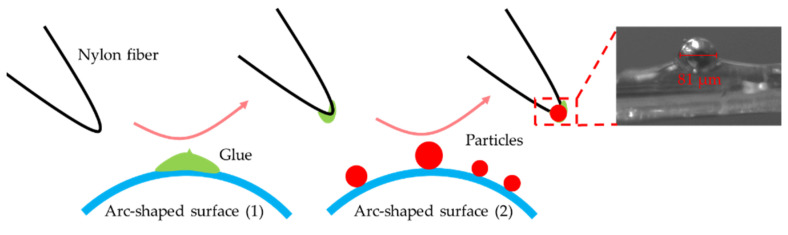
Preparation process of experimental samples. Through the observation and measurement under a microscope, the morphology of the particles was approximately spherical in shape, and the size was about 81 μm. In addition, the particle and fiber bonding can achieve accurate control of the velocity.

The samples used in this experiment were ferromagnetic metal particles. Their morphology and size were observed and measured under a Nikon SMZ1270 stereo microscope (Nikon Corporation, Tokyo, Japan). As shown in Figure 2, they were approximately spherical, and their size was about 81 μm. Many researchers usually prepare samples by mixing metal particles with oil, and then pushing the oil carrying particles through the sensor by a micro-injection pump. However, this method has certain shortcomings [31]. Firstly, it cannot achieve accurate control of the flow velocity of metal particles, since it directly controls the rotational speed of the micro-injection pump to achieve the control of the oil flow velocity. However, due to the irregularly distributed particles in the oil, their flow velocity is not equal to the oil velocity. Secondly, due to the influence of the oil liquid, the size of metal particles measured using this scheme is not accurate enough.

In our work, particles were bonded to nylon fibers. The advantages of this method are as follows: Firstly, the velocity of the nylon fibers and particles is synchronous. We can control the velocity of the particles accurately by controlling the nylon fibers. Secondly, the particles are bonded to the fibers after accurate measurement, which can ensure the accuracy of the measured particle size. Finally, the particles can be used repeatedly, which could eliminate the influence of samples during the experiments. The specific steps for sample preparation were as follows: As Figure 2 shows, the nylon fiber was firstly dipped into a small amount of glue on an arc-shaped surface. Then, a particle of the desired size on another arc-shaped surface was picked up by the nylon fiber with a small amount of glue. All steps above were carried out under the help of micromanipulation and a microscope.

### 3.2. Sensor Design and Fabrication

**Figure 3:**
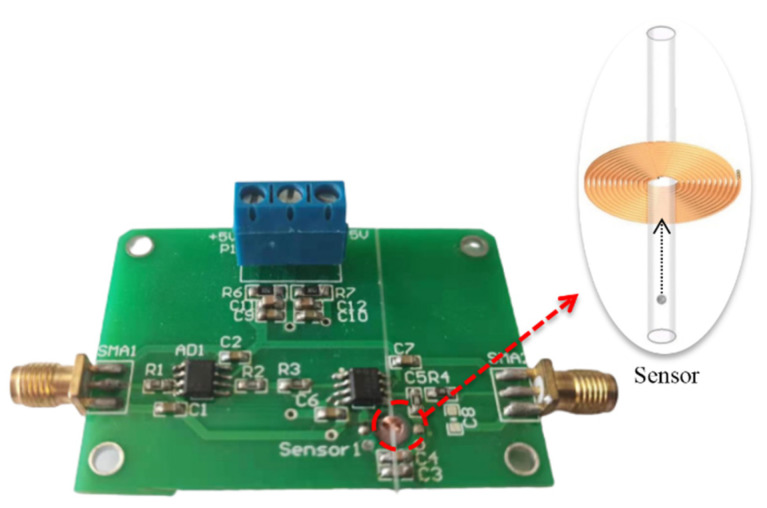
Signal detection circuit board and micro-point sensor. The sensor consists of a planar micro-inductance coil and a straight micro-channel. The micro-inductance coil is connected to capacitors to form a resonant circuit, which greatly improves the detection sensitivity.

The overall design of the sensor and the circuit is shown in Figure 3. The sensor includes a straight micro-channel and a short micro-inductance coil. In order to make the particles move as straight as possible, keep the trajectory as constant as possible, and avoid hindering the flow of particles, a micro-channel diameter of 300 microns was used. The detection sensitivity of the sensing coil increases with the increase in coil turns, but decreases with the increase in the enameled wire diameter [10]. Therefore, we used a coil with 40 turns, an inner diameter of 600 μm, and an outer diameter of 1250 μm. The number of layers was 4, the number of windings per layer was 10, and the enameled wire diameter was only 50 μm. The coil was wound by an automatic hot air winder (Dongguan Yinzhuoen Precision Automation Co., Ltd., Dongguan, China). In addition, in order to observe the effect of the particles’ velocity on the detection results more clearly, a relative long coil axial length of 260 μm was adopted. The magnetic field inside the sensing coil was radially uneven, and the magnetic field distribution at the edge of the hole in the sensing coil was larger than that at the center [53]. Therefore, the micro-channel was placed on the inner wall of the coil, so that the detection area was in the region of the maximum magnetic field intensity. Finally, we connected the induction coil with capacitors to form a resonant circuit. The information of all components constituting the sensor and detection circuit is listed in Table 1.

### 3.3. Experimental Setup

**Figure 4:**
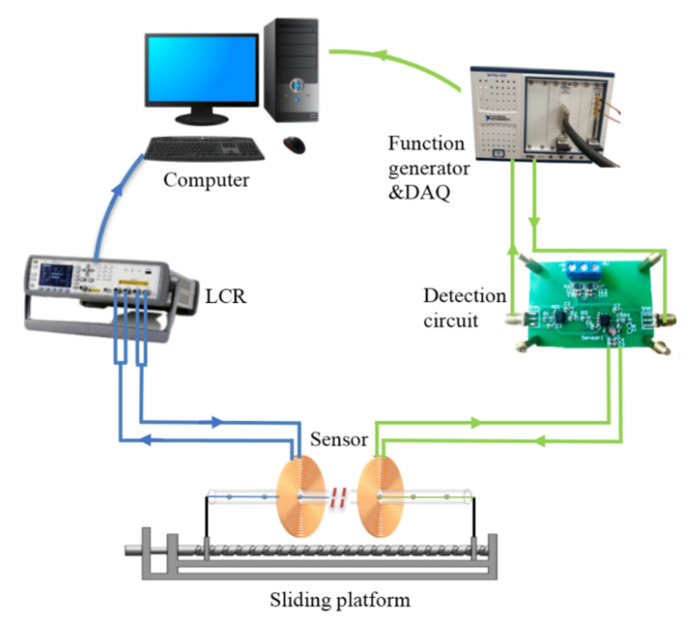
Signal detection setup of the two methods. In order to prove the effectiveness of the HFVA method, the detection signals were measured by the two methods at the same time. One signal was measured by the HFVA method, and another was measured by the LCR meter (under the same conditions).

The detection systems of the two methods are shown in Figure 4. In the HFVA method, the signal detection system included a signal detection circuit, a sensing coil, a function generator (NI PXIe-5542 arbitrary waveform generator, National Instruments, Austin, TX, USA), data acquisition equipment (NI PXI-6124 multi-function I/O module, National Instruments, Austin, TX, USA), a computer (installed with LabVIEW 2010 (National Instruments, Austin, TX, USA)), and sliding platforms. Details of all experimental elements are shown in Table 2. The function generator applied a high-frequency alternating voltage (2 MHz, 0.5 V) to the sensing coil. When the ferromagnetic debris passed through the sensing coil, the amplitude of the output voltage increased, which was then sampled by high-frequency synchronized sampling (2 MHz) and saved to the computer. Then, the saved data were further analyzed by LabVIEW software according to a certain data processing program and plotted as a pulse signal diagram output. The amplitude, polarity, and number of pulses in an output signal indicate the size, type, and concentration of particles, respectively. In the experiment, we used a fan to cool the circuit, and the temperature remained approximately constant.

In the LCR method, a sensing coil was directly connected to the LCR meter (Keysight E4980A). The LCR meter applied a certain frequency of excitation voltage (2 MHz, 0.2 V) to the sensing coil and collected the output results in “MOD” mode. Then, the collected data were transmitted to the computer for further processing and output.

The ferromagnetic debris with a diameter of 81 μm was attached to the middle of the nylon fiber by glue, so that the debris could be dragged through the sensing coil repeatedly. The two terminals of the nylon fiber were connected to the two sliding platforms tightly, which ensured that the particle maintained the same flow velocity as the sliding platform. By controlling the sliding platform to move forth and back repeatedly, the particle was thereby tested repeatedly. The particle velocities were set to be 6, 12, 31, 37, 43, 49, 56, and 62 mm/s, and the measurement was repeated five times at each velocity. The other parameters—temperature, particle size, voltage of the excited signal, and frequency of the excited signal—remained constant. Finally, the average value of five measurement results was calculated to eliminate the influence of accidental errors as much as possible.

## 4. Experimental Results

**Figure 5:**
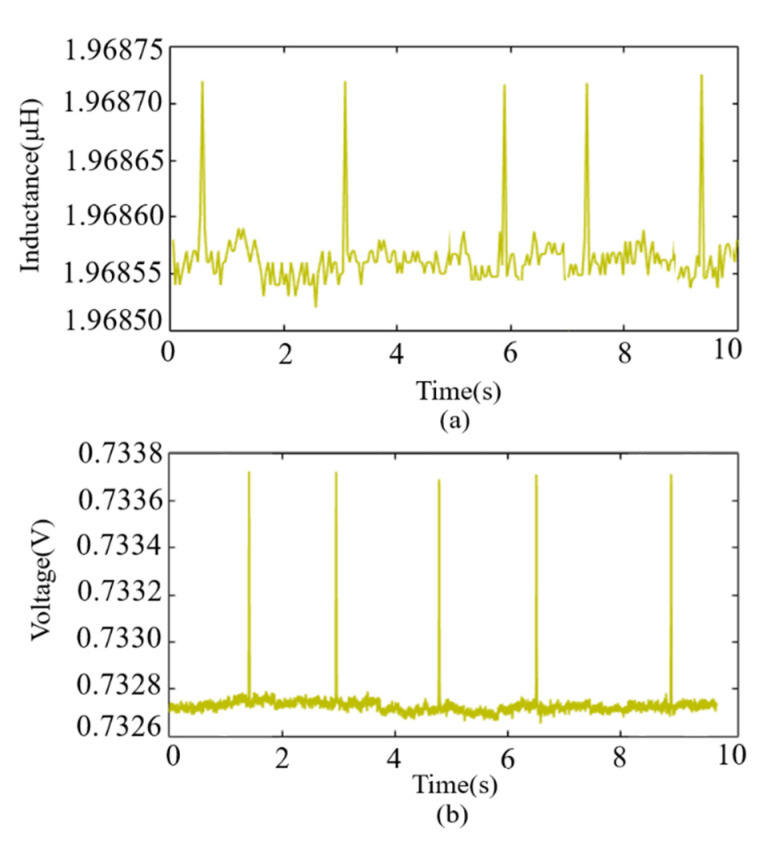
Pulse signals at a flow velocity of 49 mm/s. (**a**) Signals detected by the LCR meter; (**b**) signals detected by the HFVA method. The signal amplitude remained basically unchanged under repeated measurements, indicating that the test results are very reliable.

Figure 5a,b show the five measurement results of the iron particle at a velocity of 49 mm/s with the two methods, respectively. In Figure 5a, the signal was obtained using the LCR method. The maximum amplitude of the pulse in five repeated measurements was 1.96873 μH, while the minimum was 1.96872 μH. The maximum deviation of the signal pulses was 1 × 10^−5^ μH. The standard deviation was 0.00837 nH.

In Figure 5b, the signal was detected with the HFVA method. The maximum pulse amplitude of five repeated measurements was 0.73373 V, and the minimum value was 0.73369 V. The maximum deviation of the signal pulses was 0.4 × 10^−3^ V. The standard deviation was 0.01351 mV. The results of the repeated experiments in each group vary very little in pulse amplitude, which indicates that the experiment is repeatable.

**Figure 6:**
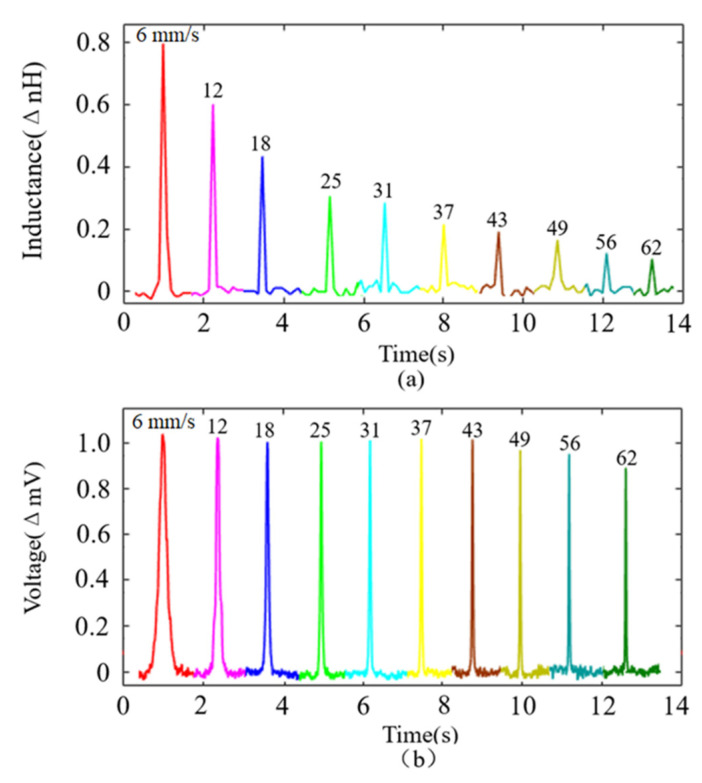
Pulse signal variation at different velocities. (**a**) Signals detected by the LCR meter; when the debris velocity increased from 6 mm/s to 62 mm/s, the pulse amplitude decreased by 85.37%. (**b**) Signals detected by the HFVA method; when the debris velocity increased from 6 mm/s to 62 mm/s, the pulse amplitude decreased by 12.75%.

In order to compare the change trend of the pulse amplitude with the increase in velocity under the two methods more directly, the pulse amplitude under different velocities was plotted, as shown in Figure 6a,b. In Figure 6a, the signal pulses were detected by the LCR meter, whose amplitude always kept decreasing with the increase in the debris velocity. When the debris velocity increased from 6 mm/s to 62 mm/s, the pulse amplitude decreased by 0.70 nH (85.37%).

In Figure 6b, the signal pulses were detected by the HFVA meter. Its amplitude was basically stable and only slightly affected by the velocity when the particles’ velocity increased from 6 mm/s to 43 mm/s, only decreasing by 0.01 mV (0.98%). When the particles’ velocity increased from 43 mm/s to 62 mm/s, the pulse amplitude experienced a relatively large decrease, which was 0.12 mV (11.88%). Throughout the whole process, the pulse amplitude only dropped by 12.75%.

**Figure 7:**
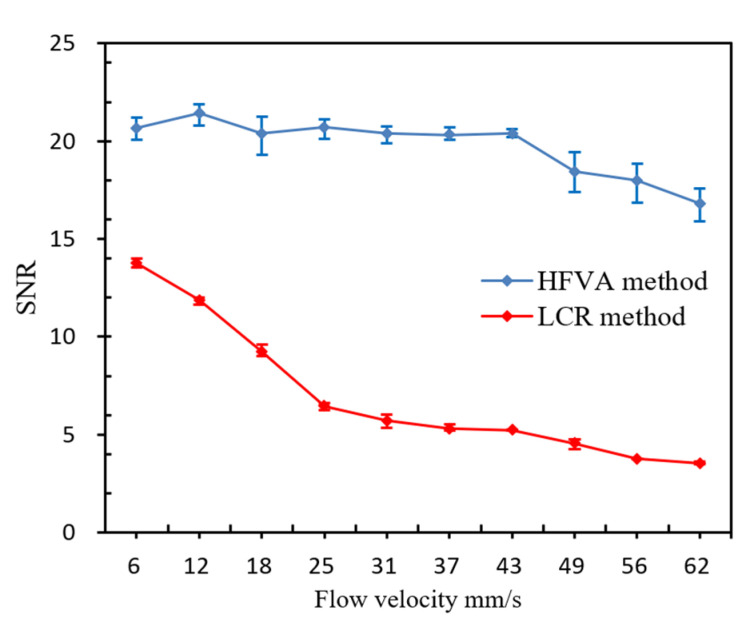
The SNR varied with the flow velocity under the two methods. With the LCR method, the SNR decreased with the increase in particle velocity. When the debris’ velocity increased from 6 mm/s to 62 mm/s, the SNR dropped by 74.36%. With the HFVA method, the SNR first remained stable with the increase in the particles’ velocity, and when the particles’ velocity rose higher than a certain value, the SNR began to slowly decrease. When the debris’ velocity increased from 6 mm/s to 62 mm/s, the SNR dropped by 21.52%.

The SNR (signal-to-noise ratio; the SNR is the amplitude divided by interference noise) of particles at different velocities is plotted in Figure 7. With the LCR method, the SNR decreased with the increase in particle velocity. When the debris’ velocity increased from 6 mm/s to 62 mm/s, the SNR dropped by 74.36%, from 13.77 to 3.53. This is consistent with the experimental result of Wang et al. [36]. This is mainly caused by two reasons. On the one hand, when the particle passes through at high velocity, the inductance of the sensing coil will suddenly increase. However, the low sampling frequency of the LCR meter means the maximum of the inductance cannot be accurately recorded. On the other hand, because of the low sensitivity of the LCR meter, it cannot track the instantaneous change in the inductance in time. Finally, due to the above two reasons, the SNR decreases continuously when the velocity increases.

However, with the HFVA method, the SNR first remained stable with the increase in the particles’ velocity, and when the particles’ velocity was higher than a certain value, the SNR began to slowly decrease. When the particles’ velocity increased from 6 mm/s to 43 mm/s, it merely had no influence on the SNR. During this process, the maximum SNR was 21.42, the minimum was 20.32, and the maximum decrease was only 5.14%. When the velocity increased from 43 mm/s to 62 mm/s, the SNR began to decrease slightly, by only 17.52%, from 20.38 to 16.81. These results indicate that, with the increasing velocity of the debris, the internal eddy current effect is gradually enhanced, and the decrease in the coil magnetic field density is continuously enhanced. Accordingly, the increase in the inductance of the coil is gradually reduced, which will eventually lead to the continuous decrease in the amplitude of the pulse signal. 

## 5. Discussion

In this study, we investigated the influence of wear debris velocity on the pulse signal of inductive detection and proposed a novel high-velocity debris detection method, which can effectively reduce the influence of the debris velocity on the detection signal. As shown in Figure 7, the SNR of the debris pulse signal decreased with the increase in the debris velocity. However, the decrease in the SNR under the novel method was significantly less than that under the LCR method. With the novel method, the maximum change in the SNR was only 5.14% with the debris’ velocity increasing from 6 mm/s to 43 mm/s. This indicates that the SNR could maintain a relatively high level at a high debris velocity. Only when the debris’ velocity was greater than 43 mm/s did the SNR begin to decrease obviously. Table 3 shows the information adopted in the research experiments of some related studies.

As shown in Table 3, the velocity used in most experimental tests is below 10 mm/s, which is far less than 43 mm/s. Thus, the proposed detection method can achieve accurate measurement of wear debris at a relatively high velocity.

## 6. Conclusions

In this work, we proposed a novel method (HFVA) for detecting high-velocity wear debris and investigated the influence of the debris’ velocity on the particle pulse. We tested the debris pulse signal with the velocity ranging from 6 mm/s to 62 mm/s. The experimental results show that the HFVA method can greatly reduce the influence of the debris’ velocity on the detection signal and achieve an accurate measurement of high-velocity wear debris. When the debris’ velocity changed within the range of 6 mm/s to 43 mm/s, the maximum relative decrease in the SNR was only 5.14%, which is almost unchanged. However, under the LCR meter method, the maximum relative decrease in the SNR was 53.15% (Figure 7). Therefore, the HFVA method can effectively reduce the impact of the debris’ velocity on the detection signal, which would benefit the efficient and accurate detection of wear debris in a high-velocity flow state. However, the HFVA method has its own limitations. The detection of high-velocity debris by the HFVA method is based on higher-frequency sampling equipment and a more stable voltage signal output. This means that the requirements of this method for equipment and circuits are higher, the detection cost will become higher, and the production process will be more complex. In future research, we will further study and optimize the resonant circuit, and try to detect high-velocity non-ferromagnetic debris. This work provides a reliable technical method for the high-velocity detection of the oil condition of equipment, and effectively improves the efficiency of oil detection. Thus, it can acquire the diagnosis and early warning signs of mechanical equipment failure more promptly.

## Figures and Tables

**Table 1:** Information table of all components constituting the sensor and detection circuit.

Elements	Specifications	Parameters
Inductance coil	Coil turns	40 turns
	Internal diameter	600 μm
	Outer diameter	1250 μm
	Axial length	260 μm
	Internal resistance (R_S_)	1.06 Ω
	Coil inductance (L_S_)	1.97 μH
Copper enameled wire	Diameter	50 μm
Resistance	R	150 Ω
Capacitance	Resonant capacitor (C_P_)	3.30 nf
Operational amplifier	AD8045	-

**Table 2:** Information table of main experimental equipment.

Elements	Specifications
Function generator	NI PXIe-5542
DAQ	NI PXI-6124
LCR meter	Keysight E4980A
Computer	Installed with LabVIEW
Sliding platform	57 stepping motor
Nylon fiber	Diameter 100 μm
Iron debris	Diameter 81 μm

**Table 3:** Experimental results of some related studies.

Debris Size (μm)	Velocity (mm/s)	SNR	Ref.
120–125	2.5	40.00	Ma et al. [10]
70	7.1	28.00	Bai et al. [16]
100–110	9.1	3.74	Shi et al. [54]
100–110	9.4	11.50	Zhang et al. [31]
48–53	9.4	5.70	Zeng et al. [55]
81	43	20.38	This work

## Data Availability

Not applicable.

## References

[1] Ren Y.J., Li W., Zhao G.F., Feng Z.H. (2018). Inductive Debris Sensor Using One Energizing Coil with Multiple Sensing Coils for Sensitivity Improvement and High Throughput. Tribol. Int..

[2] Wu T., Wu H., Du Y., Peng Z. (2013). Progress and Trend of Sensor Technology for On-Line Oil Monitoring. Sci. China Technol. Sci..

[3] Wang M., Qin G., Chen J., Liao Y. Design of Vibration Monitoring and Fault Diagnosis System for Marine Diesel Engine. Proceedings of the 2020 11th International Conference on Prognostics and System Health Management (PHM-2020 Jinan).

[4] Du T., Zuo X., Dong F., Li S., Mtui A.E., Zou Y., Zhang P., Zhao J., Zhang Y., Sun P. (2021). A Self-Powered and Highly Accurate Vibration Sensor Based on Bouncing-Ball Triboelectric Nanogenerator for Intelligent Ship Machinery Monitoring. Micromachines.

[5] Ge T. Research on Marine Engine Fault Diagnosis Based on Vibration Signal Analysis. Proceedings of the 2016 International Conference on Artificial Intelligence and Engineering.

[6] AlShorman O., Alkahatni F., Masadeh M., Irfan M., Glowacz A., Althobiani F., Kozik J., Glowacz W. (2021). Sounds and Acoustic Emission-Based Early Fault Diagnosis of Induction Motor: A Review Study. Adv. Mech. Eng..

[7] Touret T., Changenet C., Ville F., Lalmi M., Becquerelle S. (2018). On the Use of Temperature for Online Condition Monitoring of Geared Systems—A Review. Mech. Syst. Signal Process..

[8] Wu J.-D., Huang C.-K. (2011). An Engine Fault Diagnosis System Using Intake Manifold Pressure Signal and Wigner–Ville Distribution Technique. Expert Syst. Appl..

[9] Du Z., Jin X., Yang Y. (2009). Fault Diagnosis for Temperature, Flow Rate and Pressure Sensors in VAV Systems Using Wavelet Neural Network. Appl. Energy.

[10] Ma L., Shi H., Zhang H., Li G., Shen Y., Zeng N. (2020). High-Sensitivity Distinguishing and Detection Method for Wear Debris in Oil of Marine Machinery. Ocean. Eng..

[11] Du L., Zhe J., Carletta J., Veillette R., Choy F. (2010). Real-Time Monitoring of Wear Debris in Lubrication Oil Using a Microfluidic Inductive Coulter Counting Device. Microfluid. Nanofluid..

[12] Du L., Zhe J. (2013). An Integrated Ultrasonic–Inductive Pulse Sensor for Wear Debris Detection. Smart Mater. Struct..

[13] Du L., Zhe J. (2011). A High Throughput Inductive Pulse Sensor for Online Oil Debris Monitoring. Tribol. Int..

[14] Ma L., Xu Z., Zhang H., Qiao W., Chen H. (2019). Multifunctional Detection Sensor and Sensitivity Improvement of a Double Solenoid Coil Sensor. Micromachines.

[15] Cao W., Dong G., Xie Y.-B., Peng Z. (2018). Prediction of Wear Trend of Engines via On-Line Wear Debris Monitoring. Tribol. Int..

[16] Bai C., Zhang H., Zeng L., Zhao X., Ma L. (2020). Inductive Magnetic Nanoparticle Sensor Based on Microfluidic Chip Oil Detection Technology. Micromachines.

[17] Henneberg M., Eriksen R.L., Jørgensen B., Fich J. (2015). A Quasi-Stationary Approach to Particle Concentration and Distribution in Gear Oil for Wear Mode Estimation. Wear.

[18] Xiao H., Wang X., Li H., Luo J., Feng S. (2019). An Inductive Debris Sensor for a Large-Diameter Lubricating Oil Circuit Based on a High-Gradient Magnetic Field. Appl. Sci..

[19] Brendlé M.C., Diss P.H., Spano F.J. (1999). 3D Optical-Profilometric Assessment of Transfer and Its Significance for the Mechanisms of Primary Particle Detachment and Wear. Wear.

[20] Mabe J., Zubia J., Gorritxategi E. (2017). Photonic Low Cost Micro-Sensor for in-Line Wear Particle Detection in Flowing Lube Oils. Sensors.

[21] Wu T., Wu H., Du Y., Kwok N., Peng Z. (2014). Imaged Wear Debris Separation for On-Line Monitoring Using Gray Level and Integrated Morphological Features. Wear.

[22] Yan L., YouBai X. (1997). Advances in Research on a Multi-Channel on-Line Ferrograph. Tribol. Int..

[23] Appleby M., Choy F.K., Du L., Zhe J. (2013). Oil Debris and Viscosity Monitoring Using Ultrasonic and Capacitance/Inductance Measurements: Oil Debris and Viscosity Monitoring Using Ultrasonic and Capacitance. Lubr. Sci..

[24] Xu C., Zhang P., Wang H., Li Y., Lv C. (2015). Ultrasonic Echo Waveshape Features Extraction Based on QPSO-Matching Pursuit for Online Wear Debris Discrimination. Mech. Syst. Signal Process..

[25] Murali S., Xia X., Jagtiani A.V., Carletta J., Zhe J. (2009). Capacitive Coulter Counting: Detection of Metal Wear Particles in Lubricant Using a Microfluidic Device. Smart Mater. Struct..

[26] Murali S., Jagtiani A.V., Xia X., Carletta J., Zhe J. (2009). A Microfluidic Coulter Counting Device for Metal Wear Detection in Lubrication Oil. Rev. Sci. Instrum..

[27] Zhu X., Zhong C., Zhe J. (2017). Lubricating Oil Conditioning Sensors for Online Machine Health Monitoring—A Review. Tribol. Int..

[28] Flanagan I.M., Jordan J.R., Whittington H.W. (1990). An Inductive Method for Estimating the Composition and Size of Metal Particles. Meas. Sci. Technol..

[29] Liu L., Chen L., Wang S., Yin Y., Liu D., Wu S., Liu Z., Pan X. (2019). Improving Sensitivity of a Micro Inductive Sensor for Wear Debris Detection with Magnetic Powder Surrounded. Micromachines.

[30] Whittington H.W., Flynn B.W. (1995). Improved Transducer Design for Machine Wear Debris Monitoring. Electron. Lett..

[31] Zhang H., Zeng L., Teng H., Zhang X. (2017). A Novel On-Chip Impedance Sensor for the Detection of Particle Contamination in Hydraulic Oil. Micromachines.

[32] Hong W., Cai W., Wang S., Tomovic M.M. (2018). A Review for Mechanical Wear Debris Feature, Detection and Diagnosis. Chin. J. Aeronaut..

[33] Han L., Hong W., Wang S. The Key Points of Inductive Wear Debris Sensor. Proceedings of the 2011 International Conference on Fluid Power and Mechatronics.

[34] Jia R., Ma B., Zheng C., Wang L., Ba X., Du Q., Wang K. (2018). Magnetic Properties of Ferromagnetic Particles under Alternating Magnetic Fields: Focus on Particle Detection Sensor Applications. Sensors.

[35] https://hal.archives-ouvertes.fr/hal-03597258/file/feng2021.pdf.

[36] Wang Q., Zhang H., Liu E., Sun Y., Chen H. Research on the Influence of Velocity on Particle Counting Sensitivity of Microfluidic Oil Detection Chip. Proceedings of the 2015 12th IEEE International Conference on Electronic Measurement & Instruments (ICEMI).

[37] Wang C., Bai C., Yang Z., Zhang H., Li W., Wang X., Zheng Y., Ilerioluwa L., Sun Y. (2022). Research on High Sensitivity Oil Debris Detection Sensor Using High Magnetic Permeability Material and Coil Mutual Inductance. Sensors.

[38] Du L., Zhu X., Han Y., Zhe J. (2013). High Throughput Wear Debris Detection in Lubricants Using a Resonance Frequency Division Multiplexed Sensor. Tribol. Lett..

[39] Wu S., Liu Z., Yuan H., Yu K., Gao Y., Liu L., Pan X. (2019). Multichannel Inductive Sensor Based on Phase Division Multiplexing for Wear Debris Detection. Micromachines.

[40] Shi H., Zhang H., Ma L., Rogers F., Zhao X., Zeng L. (2021). An Impedance Debris Sensor Based on a High-Gradient Magnetic Field for High Sensitivity and High Throughput. IEEE Trans. Ind. Electron..

[41] Becker A., Abanteriba S., Forrester D. (2015). Determining Inductive Sensor Wear Debris Limits for Rolling Contact Fatigue of Bearings. Proc. Inst. Mech. Eng. Part J J. Eng. Tribol..

[42] Ren Y.J., Zhao G.F., Qian M., Feng Z.H. (2019). A Highly Sensitive Triple-Coil Inductive Debris Sensor Based on an Effective Unbalance Compensation Circuit. Meas. Sci. Technol..

[43] Du L., Zhe J. (2012). Parallel Sensing of Metallic Wear Debris in Lubricants Using Undersampling Data Processing. Tribol. Int..

[44] Wang M., Shi H., Zhang H., Huo D., Xie Y., Su J. (2020). Improving the Detection Ability of Inductive Micro-Sensor for Non-Ferromagnetic Wear Debris. Micromachines.

[45] Geng M., Xu T. (2017). The Lubrication Oil Wearing Particles Monitoring System with Three-Coil Inductive Sensor. DEStech Trans. Eng. Technol. Res. April.

[46] Qian M., Ren Y.J., Feng Z.H. (2020). Interference Reducing by Low-Voltage Excitation for a Debris Sensor with Triple-Coil Structure. Meas. Sci. Technol..

[47] Dumbrava V., Svilainis L. (2007). The Automated Complex Impedance Measurement System. Electron. Electr. Eng..

[48] McKinnon D.L., Smolleck H.A. (2004). Influence of Rotor Residual Flux on the Measurement of Inductance and Its Possible Use as an Impending Fault Indicator.

[49] Dorina P., Anca P. (2011). Method for Synchronized Sampling of Analog Inputs Recommended for High Speed Data Acquisition Systems. J. Electr. Electron. Eng..

[50] Noro Y., Kuno K. (2007). Synchronous Averaging for Asynchronous Sampling Data. Elect. Eng. Jpn..

[51] Song K., Cao G., Yang J., Cao P. (2012). A High-Precision Synchronous Sampling Approach for Large-Scale Distributed Wire Sensor Networks in Seismic Data Acquisition Systems. Instrum. Sci. Technol..

[52] Zhu X., Du L., Zhe J. (2017). A 3×3 Wear Debris Sensor Array for Real Time Lubricant Oil Conditioning Monitoring Using Synchronized Sampling. Mech. Syst. Signal Process..

[53] Liu E., Zhang H., Wang Q., Fu H., Chen H., Sun Y. Research on the Influence of Different Microchannel Position on the Sensitivity of Inductive Sensor. Proceedings of the 2015 12th IEEE International Conference on Electronic Measurement & Instruments (ICEMI).

[54] Shi H., Zhang H., Ma L., Zeng L. (2019). A Multi-Function Sensor for Online Detection of Contaminants in Hydraulic Oil. Tribol. Int..

[55] Zeng L., Yu Z., Zhang H., Zhang X., Chen H. (2018). A High Sensitive Multi-Parameter Micro Sensor for the Detection of Multi-Contamination in Hydraulic Oil. Sens. Actuators A Phys..

